# Knowledge sharing in infection prevention in routine and outbreak situations: a survey of the Society for Healthcare Epidemiology of America Research Network

**DOI:** 10.1186/s13756-017-0237-5

**Published:** 2017-08-08

**Authors:** Rami Sommerstein, Sonja Geser, Andrew Atkinson, Franziska Tschan, Daniel J. Morgan, Jonas Marschall

**Affiliations:** 1 Department of Infectious Diseases, Bern University Hospital, University of Bern, Freiburgstrasse 18, 3010 Bern, CH Switzerland; 2 Institute of Work and Organizational Psychology, University of Neuchatel, Neuchatel, Switzerland; 3 VA Maryland Health Care System, University of Maryland School of Medicine, Baltimore, Maryland USA

**Keywords:** Knowledge sharing, Infection prevention, Web-based training, Communication, Knowledge translation

## Abstract

In this cross-sectional Society for Healthcare Epidemiology of America Research Network survey on knowledge sharing in infection prevention we identified a rudimentary understanding of how to communicate and share knowledge within healthcare institutions. Our data support the need of further research in this important field.

**Electronic supplementary material:**

The online version of this article (doi:10.1186/s13756-017-0237-5) contains supplementary material, which is available to authorized users.

## Introduction

The recent outbreak of Ebola Virus Disease demonstrated that emerging infections may require rapid establishment, adaptation and upscaling of precaution measures. Defining state-of-the-art measures is only the first step; healthcare institutions need to manage knowledge in order to implement measures. This includes recognizing important information, retaining and sharing the information within the organization and assuring that the knowledge is used for appropriate actions [1, 2]. This crucial process is often incomplete and communication strategies related to infection prevention (IP) in hospitals are often ineffective [1, 3, 4]. Evaluating strategies to share knowledge has been overlooked in much of the healthcare epidemiology literature. There are some successful examples of improved inter-facility knowledge translation methods [5, 6]. In one case, an intervention program led to a measurable decrease in infections [6]. This experience across 1 U.S. state demonstrates that the exchange of guidelines and implementation strategies among healthcare institutions and, particularly, infection preventionists can be fruitful. Correspondingly, our study intended to gather data on current forms of knowledge sharing in IP in hospitals and between different institutions. We evaluated routine and outbreak situations and tried to identify suitable strategies for improvement.

## Methods

On February 11, 2016, a cross-sectional electronic survey was sent out by the Society for Healthcare Epidemiology of America Research Network to the IP contacts of all 228 participating institutions; this was followed by an email reminder on March 10, 2016. The survey covered different aspects of knowledge sharing: existence and availability of guidelines; means of, experience with, and obstacles towards training of HCW; feedbacks; web-based training; and education of newly employed HCW. For most questions, a routine scenario (example: hand hygiene) was compared to an outbreak scenario (example: recent Ebola outbreak).

The difference between scenarios was evaluated with χ^2^ and Mann-Whitney U tests, as indicated.

## Results

**Fig. 1:**
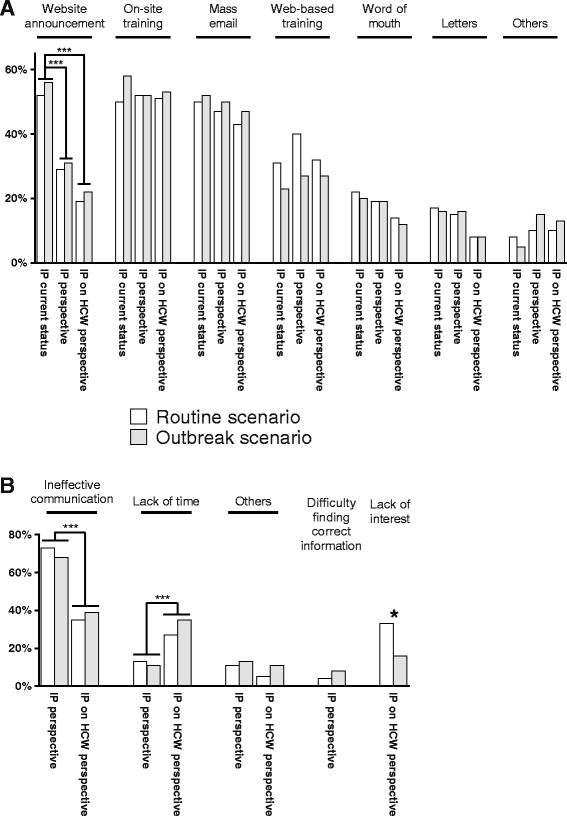
Methods for and obstacles against disseminating training updates. Frequencies of positive responses are shown for methods of sharing training updates (**a**) and on obstacles towards disseminating training updates (**b**). Multiple responses were allowed. Differences between responses on routine and outbreak scenarios are marked with an asterisk on the columns. Differences of cumulative (routine and outbreak) responses between questions are marked with a bracket. Level of significance (adjusted for multiple comparisons): * = *p* < 0.05; ** = *p* < 0.005; *** = *p* < 0.001. Questions and number of valid responses for the routine and outbreak scenarios. **a** How do you share training updates? (= IP current status); Routine *n* = 64; Outbreak *n* = 64. In your opinion, what are the most effective ways to distribute training updates? (= IP perspective); Routine *n* = 62; Outbreak *n* = 62. Please indicate what you believe are the preferred ways for healthcare workers to receive training updates? (= IP on HCW perspective); Routine *n* = 63; Outbreak *n* = 60. **b** What do you believe is the main obstacle for disseminating training updates? (= IP perspective); Routine *n* = 55; Outbreak *n* = 53. What do you believe is the main obstacle for healthcare workers’ acquisition of knowledge from training updates? (= IP on HCW perspective ); Routine *n* = 63; Outbreak *n* = 57

We received 69 valid responses from 228 institutions (30%). Of these, 47 (68%) were located in the United States, 6 (9%) in Canada, and 16 (23%) were outside North America. The full results of the survey are available online (Additional file 1). Most institutions generated hospital-specific internal guidelines (96% for routine and 93% for outbreak scenarios). Over 50% of institutions depended on one of four outside sources for preparing their instructions. The main sources were the CDC, SHEA, WHO, and the Association for Professionals in Infection Control and Epidemiology. While 70% of respondents would be willing to share their guidelines with other hospitals for free, only 30% of institutions used internal guidelines from other hospitals. The main forms of educating HCW included on-site training, mass email, website announcements, and web-based training. The approach to educate HCW did not differ between routine and outbreak scenarios. Nearly half of the responders estimated that both on-site training and mass email are the most effective ways to distribute updates. This was followed by web-based training (~35%) and website announcements (~30%). Responders thought that less frequent distribution of website announcements would satisfy the needs of IP (*p* < 0.001) and HCW (*p* < 0.001). On the contrary, responders estimated that current use of mass email and on-site training is congruent with the needs of IP and HCW (Fig. 1a). Responders considered “ineffective communication” as the main obstacle in educating HCW (Fig. 1b). They rated this obstacle as being more relevant from the IP perspective than for the receiving HCW (*p* < 0.001). In contrast, they thought that “lack of time” was more important as an obstacle for the HCW than for IP (*p* < 0.001). Lack of interest was another barrier, seen from the HCW’s standpoint, and was estimated to occur more frequently in the routine (33%) compared to outbreak (16%) scenario (*p* = 0.03). Nearly half of the responders did not test the HCWs knowledge acquisition for assessing the effectiveness of their communication strategy.

The frequency of HCW feedback on training updates was low for outbreak scenarios (20%, IQR 5–50%) and even lower for routine scenarios (5%, IQR 1–20%; *p* = 0.02). Over 40% of responders did not test HCW knowledge after training. Nevertheless, most responders regarded feedback from HCWs as helpful (routine: 66%; outbreak: 80%).

Web-based training methods were used by 70% of responders in routine scenarios and the experience was described as positive. The majority of other participants declared an interest in introducing such trainings. Web-based training methods for knowledge sharing were used more frequently in routine (70%) than in outbreak (48%) scenarios (*p* = 0.01).

For newly employed HCWs, 77% of responders’ institutions exclusively relied on information provided on the first day of employment; however only 26% thought that this was sufficient.

## Discussion

Several difficulties surrounding knowledge sharing in IP were identified by the responders of this Society for Healthcare Epidemiology of America Research Network survey: Key problems were the ineffective communication between IP and HCWs and the suspected lack of time and interest by HCWs. It is of concern that mass emails were rated among the most effective ways to distribute updates when in reality many mass emails probably go unread. Likewise, ~45% of respondents did not test HCWs knowledge acquisition for assessing the effectiveness of their communication strategy. Another obstacle was the lack of frequent and useful feedback. Thus, if an institution would like to improve knowledge sharing, this survey suggests they should first tackle ineffective intra-institutional communication. Recommendations include the use of multiple communication channels and messages that directly translate into actions [4]. The perceived lack of time and interest in HCW should also be addressed although little evidence is available in the medical field on how to ensure optimal learning processes in busy HCWs. In addition, eliciting feedback from HCWs following any type of training they receive is crucial.

We identified only minor differences between the routine and outbreak scenarios: Feedback from HCWs after their education was considered more common for outbreak scenarios. Web-based training methods were more frequently used in routine scenarios where lack of enthusiasm for knowledge acquisition was also thought to be an important limitation. Accordingly, this suggests that approaches towards creating optimal IP knowledge sharing tools do not need to distinguish between routine and outbreak scenarios. This assertion must be confirmed next.

Knowledge exchange between hospitals should be encouraged: While >90% of institutions have hospital-specific guidelines, most institutions depended on the same extramural resources when creating their guidelines. Although most hospitals were open to the idea of sharing tools, other hospitals rarely served as the source for these materials. This finding could partly be explained by results from an earlier study where the density of knowledge sharing networks in IP was found to be poor [7].

Experiences with web-based training as a tool for knowledge sharing were good among responding institutions. Although web-based learning is unlikely to address all the challenges of medical education, it was considered a valuable addition to conventional education [8]. A recent study suggested that web-based training is cost effective and comparable to on-site training with respect to acquiring knowledge [9].

Web-based training is more accessible, convenient and flexible, particularly for busy clinicians, and therefore can be implemented more easily and administered as needed. A common repository across all issuing agencies and institutions is desirable; on one hand to always provide users with access to updates and the most recent guidelines, on the other hand in order to make the information flow more uniformly.

A special focus should be placed on knowledge sharing for new employees. Most institutions provide such information exclusively on the first day of employment. This was considered to be insufficient in this survey. We believe that ongoing knowledge sharing and training is crucial to guarantee the workforce stays well-informed.

A limitation of this study was that respondents were only IP and not non-IP HCW. Therefore, the non-IP HCW perspective expressed in this study was only an estimation and may not reflect reality. Also, the 30% response rate of participating institutions of the Society for Healthcare Epidemiology of America Research Network was relatively low and therefore a participation bias cannot be excluded. Finally, only one example was chosen for each of the two scenarios, and this may not be representative for the spectrum of possible constellations.

In conclusion, we identified a rudimentary understanding of how to communicate and share IP knowledge within healthcare institutions. Our data support the need of further research in this important field.

## Additional file

Additional file 1: Knowledge Sharing in Infection Prevention in Routine and Outbreak Situations: A Survey of the Society for Healthcare Epidemiology of America Research Network. (DOCX 81 kb)

## Abbreviations

HCW: Healthcare worker
IP: Infection prevention
