# Supplementary material for: Knowledge sharing in infection prevention in routine and outbreak situations: a survey of the Society for Healthcare Epidemiology of America Research Network
Source: Antimicrob Resist Infect Control. 2017 Aug 8;6:79. doi: 10.1186/s13756-017-0237-5 (PMC5549374; doi:10.1186/s13756-017-0237-5)
Supplement: Additional file 1: — Knowledge Sharing in Infection Prevention in Routine and Outbreak Situations: A Survey of the Society for Healthcare Epidemiology of America Research Network. (DOCX 81 kb) [file 13756_2017_237_MOESM1_ESM.docx]

**Supplementary Material**

**Knowledge Sharing in Infection Prevention in Routine and Outbreak Situations:** [**A Survey of the Society for Healthcare Epidemiology of America Research Network**](https://www.ncbi.nlm.nih.gov/pubmed/26961763)

**Methods (extended version)**

Cross-sectional study in form of an electronic survey sent out by the SHEA Research Network to medical centers participating in that network. The survey management tool by Real Magnet (Bethesda, MD) was used.

Definite survey questions were elaborated together with the SHEA Research Network. The initial versions of the survey was evaluated and improved by testers of the SHEA Research Network and by healthcare epidemiologists at Bern University Hospital.

The survey was voluntary and consisted of 30 questions.

Audience: This survey was intended for those in roles responsible for disseminating infection prevention information, e.g. the hospital epidemiologist or infection preventionist or others with this responsibility.

Definitions: For the project, "knowledge sharing" was defined as the acquisition and dissemination of new and updated guidelines, fact sheets, and written information within the institution.

The survey was sent out on February 11, 2016, followed by a reminder on March 10, 2016.

Responses to questions were either collected as single choice, multiple choice, or free text.

Data was entered into an electronic spreadsheet and analyzed with Microsoft Excel. Chi Square tests were used to explore how responses differed between groups. T-tests were used to explore means between groups.

We explored knowledge sharing for hand hygiene aspects (representing a standard scenario; STD) and for the recent EVD outbreak as an example of an emerging disease (non-standard scenario; NSTD).

**Full Results**

1. Valid Responses

- 69/228 (30%) of member institutions

1. Respondent Characteristics

- Geographic Distribution: 69/69 valid responses

- United States: 47 (68%)

- Canada: 6 (9%)

- Other: 16 (23%)

- Hospital Size (Number of Beds): 56/69 valid responses

- Hospital Type: 41/69 valid responses
 56/69 valid responses

Additional information: "Other"-responses included: Rehabilitation, long term acute care hospital (LTACH)

- Hospital Size (Number of HCW): 65/69 valid responses

- Respondent Function: 69/69 valid responses

- Hospital epidemiologist: 48 (70%)

- Infection Preventionist: 14 (20%)

- Other: 7 (10%)

1. Survey Results

***Q 1.1: Do you have hospital-specific guidelines/fact sheets/written instructions for HCWs available? (single choice)***

Conclusion: Most of the hospitals dispose of specific guidelines/instructions. Responses were normally distributed between the STD and NSTD groups.

***Q 1.2: Which sources do you use for preparing hospital-specific instructions? (multiple choices and free text)***

Additional information: 21/69 answers (30%) “Other” (not listed above); Free text responses: Local, provincial, state or national guidelines

Conclusion: Most institutions depended on several sources for preparing their instructions. Only a minority used other hospitals’ input as a source. Responses were normally distributed between the STD and NSTD groups.

***Q 1.3: Are you willing to share guidelines/fact sheets/written instructions with other hospitals? (single choice and free text)***

Additional information: "Other"-responses included: prior management/team approval.

Conclusion: > 70% of participating centers are open to sharing their instructions for free. Responses were normally distributed between the STD and NSTD groups.

***Q 1.4: How do you provide HCWs with necessary guidelines/fact sheets/written instructions? (multiple choice and free text)***

Additional information: "Other"-responses included: Email, (mandatory) online education/web-based training, training session/simulation lab, special team training

Conclusion: The intranet web site is the main medium for providing instructions to HCWs. Responses were normally distributed between the STD and NSTD groups.

***Q 2.1: On average, how often do you update guidelines/fact sheets/written instructions? (single choice)***

Additional information: "Other"-responses included: as needed

Conclusion: Responses are not normally distributed between the STD and NSTD groups (p<0.001). In NSTD group, updates are offered more frequently.

***Q 2.2: How do you share updates? (multiple choice and free text)***

Additional information: "Other"-responses included: Vary from the extend of update, discussion at unit/department meeting, educational video, link nurse

Conclusion: The three main forms of distribution include mass email, on-site training and website announcements. Responses were normally distributed between the STD and NSTD groups.

***Q 2.3+2.4: Please estimate the percentage of HCWs you reach with updates to STD/NSTD guidelines/fact sheets/written instructions (free text)***

Additional information: Free text responses included: Mostly sent to 100% of the HCWs, but uncertain how many really read the emails.

Conclusion: Respondents estimated that a median of 70% of HCWs can be reached for both groups. Mean values did not differ (p=0.94)

***Q 2.5: On average, how often do you distribute updates? (single choice and free text)***

Additional information: "Other"-responses included: as needed

Conclusion: Responses are not normally distributed between the STD and NSTD groups (p=0.018). In NSTD group, updates are distributed more frequently.

***Q 2.6: In your opinion, what are the most effective ways to distribute updates? (multiple choice with maximum three answers, free text)***

Additional information: "Other"-responses included: Additional instructions regarding mass-email; no effective way, information only to target person, unit/department meeting, special team training, and skills fairs

Conclusion: Mass-email and onsite training are the most effective ways to distribute updates according to respondents. Responses were normally distributed between the STD and NSTD groups.

***Q 2.7: Please indicate what you believe are the preferred ways for HCWs to receive updates? (multiple choice with maximum three answers, free text)***

Additional information: "Other"-responses included: Additional instructions regarding mass email; unit/department meeting, personal communication

Conclusion: Mass-email and onsite training were considered the preferred ways for HCWs to receive updates according to respondents. Responses were normally distributed between the STD and NSTD groups.

***Q 2.8: What do you believe is the main obstacle for HCWs' acquisition of knowledge from updates? (single choice and free text)***

Additional information: "Other"-responses included: mainly lack of time

and anxiety/fears (NSTD group only)

Conclusion: The three main obstacles in both groups were thought to be “ineffective communication”, “no time”, and “no interest”. Responses were normally distributed between the STD and NSTD groups.

***Q 2.9: What do you believe is the main obstacle in disseminating updates? (single choice and free text)***

Additional information: "Other"-responses included: competing time demands, overload, frequently changing info, difficult to get to target audience, presenting the correct amount of information in a culturally appropriate format

Conclusion: The single most important obstacle in disseminating updates was believed to be ineffective communication. Responses were normally distributed between the STD and NSTD groups.

***Q 2.10: How do you test HCWs knowledge acquisition of updates? (multiple choice and free text)***

Additional information: "Other"-responses included: Audit, simulation, assessment

Conclusion: >40% of responding institutions do not test (even more pronounced in the NSTD group). Responses were normally distributed between the STD and NSTD groups.

***Q 2.11+2.12: Please estimate the percentage of HCWs who give feedback on knowledge acquisition of updates to guides (free text)***

Additional information: “0%” as answer 8x in STD group, and 3x in NSTD group

Conclusion: The feedback rate in both constellations was estimated to be very low. The mean feedback rates for the STD and NSTD group was estimated at 16% and 27%, respectively. Feedback rate were lower in the STD group (p=0.04).

***Q 2.13: In general, do you consider feedback that you receive helpful? (single choice and free text)***

Additional information: Remarks made by participant: to address barriers to implementation; refine future updates of guidelines or the development of new guidelines and resource sheets; to improve content and methods; to improve teaching style; feasibility for practice; communications is understood; show better compliance

Conclusion: Feedbacks are considered helpful. Responses were normally distributed between the STD and NSTD groups.

***Q 3.1: Do you use web-based training methods for information dissemination? (single choice)***

Conclusion: Responses were not normally distributed between the STD and NSTD groups (p=0.045). Web-based training is more frequently used in the STD group.

***Q 3.2: If yes, please describe your experience using web-based training (single choice)***

Conclusion: > 50% of the participants described their experience with web-based training as good or excellent. Responses were normally distributed between the STD and NSTD groups.

***Q 3.2: If no, would you be willing to use web-based training methods? (single choice)***

Conclusion: >75% of the respondents that do not use web-based training would be willing to do so. Responses were normally distributed between the STD and NSTD groups.

***Q 3.3: Would you be interested in a common repository of web-based training methods? (single choice)***

Conclusion: > 80% of the participants would favor a common repository. Responses were normally distributed between the STD and NSTD groups.

***Q 4.1: How do you share guidelines fact-sheets written instructions with HCWs new to your institution? (multiple choice and free text)***

65/69 valid responses

Additional information: "Other"-responses included: Mainly given answer was orientation course/program; less frequent answers were intranet repository, web-based/online training, licensing program or none (only 2 participants)

Conclusion: >75% of all the institutions rely for the most part on information provided on the first day of employment.

***Q 4.2: Do you think these means are sufficient for sharing information? (single choice and free text)***

65/69 valid responses

Conclusion: Only 26% think that these means are sufficient.
